# Antioxidant activity of Coridius chinensis extracts on manganese‐induced testicular damage in rats

**DOI:** 10.1002/tox.22777

**Published:** 2019-05-29

**Authors:** Qiongyou Liu, Changhuo Cen, Huihui Fu, Fengyue Wang, Yong Wang, Tiantian Xu, Xiaohui Hou

**Affiliations:** ^1^ Department of Basic Medical Sciences Zunyi Medical University Zunyi China

**Keywords:** antioxidant, apoptosis, *Coridius chinensis* extracts (CcE), manganese

## Abstract

Coridius chinensis (C. chinensis) is a traditional Chinese medicine that has been used to treat pain, erectile dysfunction, and other diseases. Our previous study demonstrated that manganese‐induced reproductive damage was partially rescued by a medium dose of C. chinensis treatment in rat. However, the underlying mechanism is unknown. In this study, we found that the weight of reproductive organs and the sperm count in manganese‐exposed rat were partially rescued by C. chinensis extracts (CcE) treatment. The number of apoptotic cells was significantly decreased and the expression of malondialdehyde, cytochrome *c*, and caspase‐3 in manganese‐exposed rats was significantly decreased after high dose of CcE treatment. Further studies revealed that the activity of superoxide dismutase, total antioxidant capacity, and glutathione peroxidase enzymes was significantly increased in testis tissues and serum of manganese‐exposed rats with high dose of CcE treatment. Taken together, the results of this study suggest that CcE inhibits the Mn^2+^‐induced apoptosis in testes by inducing the activity of antioxidants.

## 1 INTRODUCTION

Manganese (Mn^2+^) is an essential ion that is required for normal immune function, bone growth, reproduction, and blood sugar regulation,^1^ but extreme excess intake of manganese causes serious neurotoxicity, reproductive toxicity, and even death.^2^, ^3^ It has been reported that high dose of manganese exposure causes decreased testis weight, sperm concentration, and serum testosterone level in animal models. In human, high manganese level was associated with increased risk of low sperm motility and concentration.^4^ Manganese exposure also causes decreased activity of superoxide dismutase (SOD) and glutathione peroxidase (GSH‐Px), and increased apoptosis of spermatogenic cells and malondialdehyde (MDA) levels.^5^, ^6^, ^7^, ^8^, ^9^, ^10^

*Coridius chinensis* (*C. chinensis*) is a traditional Chinese medicine, which has been used to treat pain, erectile dysfunction, and kidney diseases.^11^ Our previous study found that Mn^2+^‐induced reproductive damage in rats was partially rescued by *C. chinensis* treatment. The testosterone levels and sperm concentration were increased and sexual behavior indexes including capturing time and ejaculation ability were recovered.^12^ These studies indicate that manganese exposure‐induced reproductive damage can be intervened by *C. chinensis*. In this study, we further investigate the underlying mechanism of *C. chinensis* in protecting Mn^2+^‐induced damage in rat testis. We find that *C. chinensis* extracts (CcE) inhibits the Mn^2+^‐induced apoptosis in testes by inducing the activity of antioxidants.

## 2 MATERIALS AND METHODS

### 2.1 Chemicals and reagents

MnCl_2_•4H_2_O was purchased from Shanghai Chemical Reagent Co. Ltd of China National Pharmaceutical Group Corporation (China). The MDA, total superoxide dismutase (T‐SOD), total antioxidant capacity (T‐AOC), and GSH‐Px test kits were purchased from Nanjing Jiancheng Bioengineering Institute (China).

### 2.2 *Coridius chinensis* collection and extraction

*Coridius chinensis* species was collected from Zheng'an County in Guizhou Province, China, between October and November 2014. The extraction process was performed following the instruction of *Chinese Pharmacopoeia*. In brief, *C. chinensis* species was washed with 50 °C~60 °C distilled water to remove impurities and air‐dried for 2 days. The dried *C. chinensis* was crushed with a hammer mill crusher for 30 minutes. The crushed sample (0.2 kg) was extracted with 1 L of distilled water and boiled at 95 °C~100 °C for 30 minutes twice. The soluble extract was filtered using a nylon filter. The filtrate was dried using lyophilization. The aqueous extract from *C. chinensis* was used in this study.

### 2.3 Experimental animals and treatment regime

All animal studies were carried out in accordance with the protocols approved by the Animal Research and Ethics Committees of Zunyi Medical University. All Sprague‐Dawley (SD) rats (180‐200 g) were purchased from the Third Military Medical University, Chongqing, China. The rats were supplied with standard pellet diet and tap water under a 12 hours light/dark cycle and room temperature of 22 °C‐24 °C. Fifty male SD rats were divided into five groups: one control group and four treatment groups. The control group was administered with intraperitoneal injection of normal saline (0.5 mL) and the four treatment groups were administered with intraperitoneal injection of manganese chloride (30 mg/kg BW [body weight]) containing intragastric administration of 0, 50, 100, and 200 mg/kg CcE, respectively, for 28 days, and each group (n = 10) was treated as shown in Table 1.

**Table 1:** Treatment on each group of rats

Group	Treatment	
	Days 1‐14 (intraperitoneal injection)	Days 1‐28 (via a gastric tube)
Control	Saline, 0.5 mL	Distilled water, 1 mL
Mn	Mn (30 mg/kg BW), 0.5 mL	Distilled water, 1 mL
50 CcE + Mn	Mn (30 mg/kg BW), 0.5 mL	CcE (50 mg/kg BW), 1 mL
100CcE + Mn	Mn (30 mg/kg BW), 0.5 mL	CcE (100 mg/kg BW), 1 mL
200CcE + Mn	Mn (30 mg/kg BW), 0.5 mL	CcE (200 mg/kg BW), 1 mL

*Notes*: The Mn group (positive control) is designed based on Cai et al.^13^ Days 1‐14 are the induction and preventive periods, and days 15‐28 are the cure period.

Abbreviations: BW, body weight; CcE, *Coridius chinensis* extracts; Mn, MnCl_2_•4H_2_O (water dissolved).

### 2.4 Epididymal sperm concentration

The left epididymis+vas deferens were dissected from male SD rats. Sperms were extruded from the epididymis+vas deferens and incubated in phosphate‐buffered saline (PBS) for 30 minutes at 37 °C. The incubated sperms were centrifuged (500*g*, 37 °C, 5 minutes) to wash and separate the mature sperm pellet. The sperm pellets were resuspended and diluted at 1:10 ratio with 0.3% (v/v) bovine serum albumin (BSA)‐KSOM (potassium‐enriched simplex optimized medium, Embryo Max KSOM Powdered Mouse Embryo Culture Medium; Millipore Catalogue No. R‐MR‐020P‐5D) and then transferred to a hemocytometer for counting.

### 2.5 Tissue collection and histological analysis

The testes and epididymis+vas deferens of Mn^2+^‐treated and control SD rats were dissected and weighed immediately after euthanasia on the day after termination of the Mn‐CcE coadministration. Testes were fixed in 4% (v/v) paraformaldehyde for up to 24 hours, stored in 70% (v/v) ethanol, and embedded in paraffin. The 5‐μm‐thick sections were prepared and mounted on glass slides.^14^ After deparaffinization, the sections were stained with hematoxylin‐eosin (H&E) for histological analysis.

### 2.6 Activity assays of MDA, T‐AOC, T‐SOD, and GSH‐Px in serum and testis

All male SD rats were sacrificed to expose the left ventricle of the heart. Blood was collected by puncture of the left ventricular using 1 mL of heparin to prevent blood clotting and centrifuged at 5000 rpm at 4 °C for 10 minutes to separate the serum from blood cells. The testis was homogenized in cold normal saline (tissue weight:normal saline = 1 g:9 mL), centrifuged at 2500 rpm at 4 °C for 10 minutes, and then the supernatant was collected. The activity assays of MDA, T‐AOC, T‐SOD, and GSH‐Px of the serum and testicular tissue extraction were performed with MDA assay kit, total antioxidant capacity assay kit, T‐SOD assay kit, and GSH‐Px assay kit (Nanjing Jiancheng Bioengineering Institute, China) according to the manufacturer's instructions, respectively.

### 2.7 Western blot analysis

The testis homogenate (100 μg) was separated on a 10% SDS‐PAGE gel and transferred to a methanol‐activated PVDF membrane (Millipore) by electroblotting. The membrane was then blocked with 5% nonfat milk powder in 10 mM PBS buffer (137 mM NaCl, 2.7 mM KCl, 10 mM Na_2_HPO_4_, and 2 mM KH_2_PO_4_) for 2 hours at 37 °C. The blocked membrane was then incubated with anti‐caspase‐3 monoclonal antibody (1:2000, Cell Signaling Technology Group, 9662S), anti‐cyt *c* monoclonal antibody (1:2000, Cell Signaling Technology Group, 11940S), or anti‐GAPDH (glyceraldehyde‐3‐phosphate dehydrogenase) monoclonal antibody (1:2000, Proteintech) in blocking solution (5% nonfat milk powder in 10 mM PBS) at 4 °C overnight, washed three times with Tris‐Buffered Saline and Tween20 (TBST) for 5 minutes each, and incubated with horseradish peroxidase (HRP)‐conjugated goat anti‐rabbit immunoglobulin G (IgG) (1:2000; Chemicon, Proteintech) at 37 °C for 2 hours. After washing three times, the membranes were exposed to the chemiluminescence substrate (ECL; 7Sea Biotech Co., Shanghai, China) according to the manufacturer's instructions.

### 2.8 Immunohistochemistry

After deparaffinization and rehydration, the paraffin‐embedded sections were performed using a Vectastain ABC (avidin‐biotin‐peroxidase) kit (Vector Laboratories, Burlingame, CA) as recommended and using the primary rabbit antibodies MVH (1:1000, Abcam, ab13840) and SOX9 (1:500, Millipore, AB5535), and these were followed by staining with HRP‐conjugated secondary antibody. After rinsing with PBS, the sections were stained with 3,3′‐diaminobenzidin.^15^ Images were captured using a Nikon microscope with a CCD camera.

### 2.9 Apoptosis detection

Apoptosis detection of testicular cells was conducted with the Promega DeadEnd Fluorometric TUNEL System in accordance with the manufacturer's instructions. The paraffin‐embedded testis sections were assayed by the terminal deoxynucleotidyl transferase‐mediated deoxyuridine triphosphate nick end labeling (TUNEL) method to detect internucleosomal DNA fragmentation that is characteristic of apoptosis. The green fluorescence of apoptotic cells was detected in a blue background using the Nikon microscope, and the images were captured by the Nikon DS‐Ri1 CCD camera.

### 2.10 Statistical analysis

Data were statistically analyzed by one‐way ANOVA followed by Tukey's multiple comparison test using the SPSS 19.0 software (SPSS, Inc.). Significance was set at *P* < .5.

## 3 RESULTS

### 3.1 Epididymal sperm concentrations and weights of male reproductive organs

The sperm concentration in Mn^2+^‐treated group was significantly decreased compared to that of control group (Table 2). Compared to the Mn^2+^ group, the number of sperms in 50CcE + Mn^2+^ and 100CcE + Mn^2+^ groups was slightly increased, but not significantly different. The sperm concentration in the 200CcE + Mn^2+^ group was significantly increased compared to the Mn^2+^ group (Table 2). The relative weight of the testes and epididymis+vas deferens was significantly decreased in the Mn^2+^ group compared to that of control rats. In consistent with the sperm concentration, the relative weight of the testes and epididymis+vas deferens was slightly increased in low dose CcE‐treated groups (Table 2) compared to the Mn^2+^ group, whereas it was significantly improved in the 200 mg/kg BW CcE‐treated group (Table 2).

**Table 2:** Sperm concentration and relative weights of the reproductive systems of control and experimental rats

Group	Sperm concentration (1 × 10^7^ cells/ml)	Relative weights (g/100 g body weight)	
		Testes	Epididymis+vas deferens
Control	2.76 ± 0.68	0.91 ± 0.06	0.31 ± 0.05
Mn	1.60 ± 0.31**	0.53 ± 0.03***	0.18 ± 0.01**
50CcE + Mn	2.48 ± 0.32	0.84 ± 0.08	0.23 ± 0.02
100CcE + Mn	3.01 ± 0.76	0.92 ± 0.09	0.32 ± 0.02
200CcE + Mn	4.11 ± 0.16*	1.11 ± 0.08*	0.39 ± 0.02*

*Note*: Data were expressed as mean ± SD (n = 10).

Abbreviation: CcE, *Coridius chinensis* extracts.

*significant differences (*P* < .01) compared with the Mn group; **significant differences (*P* < .05) compared with the control group, ***Significant differences (*P* < .01) compared with the control group, respectively.

### 3.2 Protective effect of CcE on Mn^2+^‐induced testicular damage

**Figure 1:**
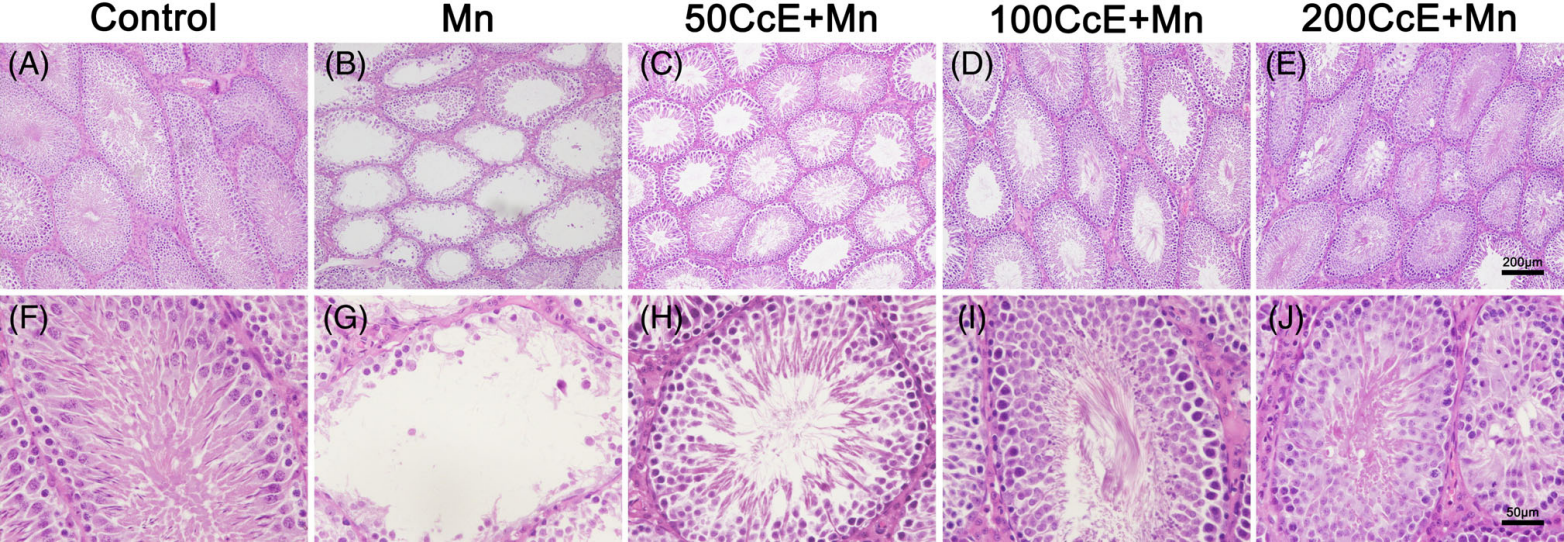
The histology of testes from control and CcE‐treated rats. A,F, Control; B,G Mn^2+^; C,H, 50CcE + Mn^2+^; D,I, 100CcE + Mn^2+^; and E,J, 200CcE + Mn^2+^ [Color figure can be viewed at wileyonlinelibrary.com]

To further investigate the effects of CcE on Mn^2+^‐induced testicular damage in SD rats, the histology of testis was examined by H&E staining. As shown in Figure 1, massive germ cell loss was observed in the seminiferous tubules of Mn^2+^‐treated rats. The Mn^2+^‐induced testicular damage included sloughing of germ cells and degeneration of early and late spermatogenic cells in CcE‐treated groups (Figure 1C‐E,H‐J). The abnormality of seminiferous tubules was also observed in the 50CcE + Mn^2+^ group (Figure 1C,H). By contrast, the germ cell loss was significantly improved in the testes of 100CcE + Mn^2+^ (Figure 1D,I) and 200CcE + Mn^2+^ (Figure 1E,J)‐treated rats. These results indicate that the Mn^2+^‐induced testicular damage was rescued by CcE administration in a dose‐dependent manner.

### 3.3 Effect of CcE on antioxidant substances

MDA is the end product of lipid peroxidation accumulated by intracellular oxidative damage. The content of MDA in the organism can indirectly reflect the intensity of lipid peroxidation in the body and the degree of free radical damage to tissue cells. The tissue and serum MDA levels were significantly increased in the Mn^2+^‐treated group compared to the control group, and it was significantly decreased in different doses of CcE‐treated groups (Tables 3 and 4). T‐AOC, T‐SOD, and GSH‐Px are antioxidant enzymes used to remove reactive oxygen species (ROS) in the body to reduce intracellular ROS and protect cells. Their activity indirectly reflects the ability of the body to scavenge oxygen free radicals. The activity of T‐AOC, T‐SOD, and GSH‐Px enzymes was significantly decreased in Mn^2+^‐treated group compared to the control group, and it was significantly increased after intragastric administration of different doses of CcE.

**Table 3:** Results of MAD, T‐AOC, T‐SOD, and GSH‐Px in testis

Group	MAD (nmol/mL)	T‐AOC (U/mL)	T‐SOD (U/mL)	GSH‐Px (U/mL)
Control	7.26 ± 0.36 b	2.25 ± 0.08 b	25.88 ± 1.75 c	122.07 ± 0.98 b
Mn	11.41 ± 0.94 d	1.83 ± 0.04 a	18.87 ± 0.95 a	103.96 ± 2.09 a
50CcE + Mn	8.13 ± 0.30 c	2.17 ± 0.04 ab	22.80 ± 1.90 b	117.53 ± 1.19 b
100CcE + Mn	7.43 ± 0.15b c	2.85 ± 0.16 c	28.88 ± 0.74 d	131.89 ± 2.78 c
200CcE + Mn	6.34 ± 0.15 a	4.45 ± 0.48 d	31.81 ± 1.67 e	177.51 ± 5.45 d

*Note*: Data were expressed as mean ± SD (n = 6), different lowercase letters behind the numbers are significantly different (*P* < .05).

Abbreviation: CcE, *Coridius chinensis* extracts.

**Table 4:** Results of MAD, T‐AOC, T‐SOD, and GSH‐Px in serum

Group	MAD (nmol/mL)	T‐AOC (U/mL)	T‐SOD (U/mL)	GSH‐Px (U/mL)
Control	5.99 ± 0.40 b	3.49 ± 0.20 b	453.40 ± 7.51 b	559.89 ± 4.15 c
Mn	8.06 ± 0.47 d	2.50 ± 0.18 a	407.82 ± 7.06 a	463.82 ± 8.69 a
50CcE + Mn	6.91 ± 0.31 c	3.44 ± 0.10 b	456.03 ± 9.39 b	506.06 ± 19.78 b
100CcE + Mn	5.89 ± 0.08 b	3.59 ± 0.20 b	476.89 ± 7.97 c	578.06 ± 7.30 c
200CcE + Mn	4.93 ± 0.41 a	4.30 ± 0.22 c	516.89 ± 8.55 d	610.60 ± 9.15 d

*Note*: Data were expressed as mean ± SD (n = 6), different lowercase letters behind the numbers are significantly different (*P* < .05).

Abbreviation: CcE, *Coridius chinensis* extracts.

### 3.4 Number of apoptotic cells decreases with *C. chinensis* treatment

**Figure 2:**
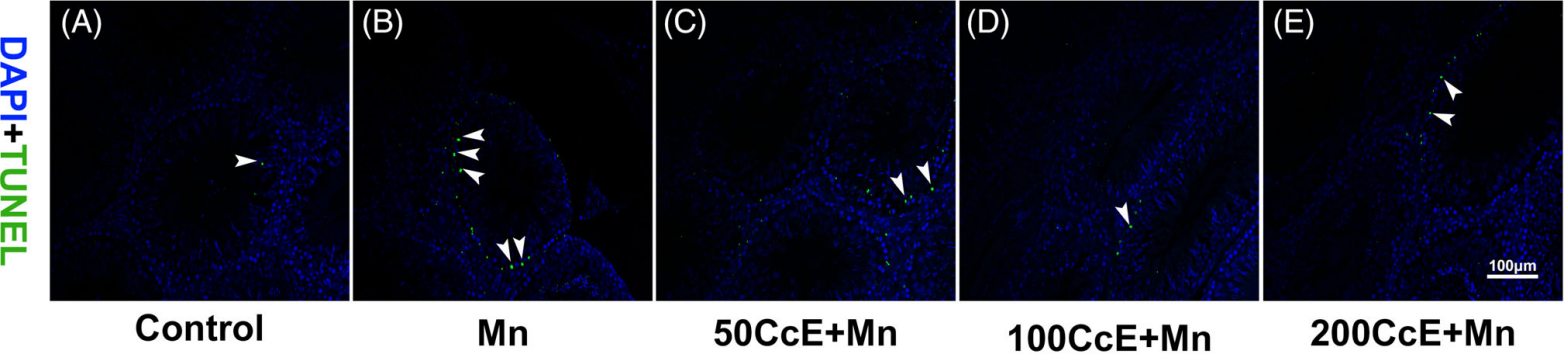
The effect of CcE on Mn^2+^‐induced cell apoptosis. A, Control; B, Mn^2+^; C, 50CcE + Mn^2+^; D, 100CcE + Mn^2+^; and E, 200CcE + Mn^2+^ [Color figure can be viewed at wileyonlinelibrary.com]

The apoptotic cells in Mn^2+^‐treated testes were examined by TUNEL assay. As shown in Figure 2, a large number of TUNEL‐positive apoptotic germ cells (Figure 2B, white arrows) were observed in the seminiferous tubules of Mn^2+^‐treated rats, and very few apoptotic cells were detected in the control group (Figure 2A, white arrow). The number of apoptotic cells (Figure 2C‐E, white arrows) was significantly decreased in Mn^2+^‐treated testes after different doses of CcE treatment.

**Figure 3:**
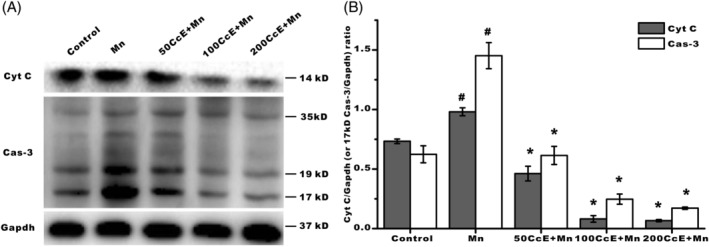
The expression of apoptosis‐associated protein was reduced after CcE treatment. CcE, *Coridius chinensis* extracts. A, Pro‐caspase‐3 (35 kD) and cleaved caspase‐3 (17 kD and 19 kD) analyzed by Western blotting. B, Cleaved caspase‐3 (17 kD) and cyt *c* analyzed by SPSS. *Significant differences (*P* < .01) compared with the Mn^2+^ group; #Significant differences (*P* < .01) compared with the control group. Data were expressed as mean ± SD (n = 6)

To further explore the potential mechanism of CcE in protecting Mn^2+^‐induced apoptosis, the expression of cytochrome *c* (cyt *c*) and caspase‐3 was examined by Western blotting analysis. The results showed that the protein level of cyt *c* and cleaved caspase‐3 was dramatically increased in the Mn^2+^‐treated group compared to that of the control group. By contrast, the expression of these two proteins was significantly reduced after administration of CcE (Figure 3).

**Figure 4:**
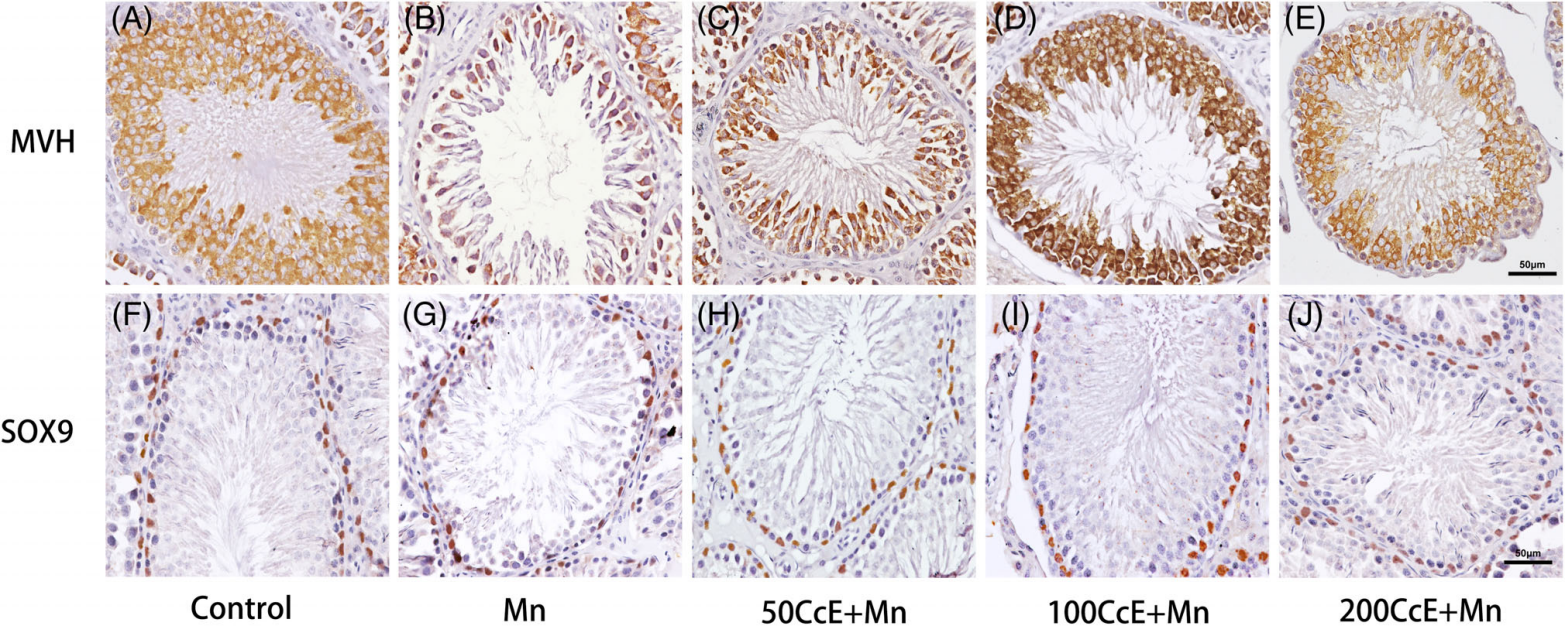
Immunohistochemistry of SOX9 and MVH. A,F, Control; B,G, Mn^2+^; C,H, 50CcE + Mn^2+^; D,I, 100CcE + Mn^2+^; and E,J, 200CcE + Mn^2+^ [Color figure can be viewed at wileyonlinelibrary.com]

To examine whether Mn^2+^ treatment affects the survival of testis somatic cells, the expression of germ cell marker MVH and sertoli cells marker gene SOX9 was examined by immunostaining. As shown in Figure 4, the number of MVH‐positive germ cells was dramatically decreased in Mn^2+^‐treated groups, whereas the number of SOX9‐positive sertoli cells was not changed. The result suggests that apoptotic cells primarily originate from germ cells.

## 4 DISCUSSION

With wide applications of Mn^2+^ and its products, it has become a major environmental pollutant that affects male reproductive health. Previous studies have shown that testis is an organ with high Mn^2+^ sensitivity, and Mn^2+^ can be stored in testicular tissues via the blood‐testosterone barrier, resulting in atrophy of testicular tissue, decrease of sperm number, and excessive apoptosis of spermatogenic cells, thereby causing pathological injury of testicles and decline of reproductive function.^16^, ^17^ And the damage caused by Mn^2+^ to male reproductive system includes the following aspects: (1) gene mutation and chromosome abnormality in spermatogenic cells^18^; (2) decreased levels of GSH‐Px, catalase (CAT), and SOD^19^; increased formation of free radicals and lipid peroxidation^20^; (3) mitochondrial damage and energy metabolism disorders; (4) upregulation of apoptotic gene *P53* and downregulation of antiapoptotic gene *BCL‐2*.^13^, ^21^ Mn^2+^ can induce apoptosis by releasing cyt *c* from the mitochondria.^22^ In this study, we found that the number of apoptotic cells and the expression of MDA, cyt *c*, and caspase‐3 were significantly increased, but the activity of SOD, T‐AOC, and GSH‐Px was significantly decreased in Mn^2+^‐exposed rats. These results indicate that oxidative stress is most likely the major reason that causes testicular damage in rat upon Mn^2+^ exposure. Our recent study demonstrated that Mn^2+^‐induced reproductive damage was partially rescued by a medium dose of *C. chinensis* treatment in rat.^12^ However, the underlying molecular mechanism is unknown. *Coridius chinensis* is an important and authentic Chinese medicinal material and has high edible and medicinal value. Previous studies have confirmed that the insect contains a variety of essential amino acids, fatty acids, vitamins, trace elements (Fe^2+^, Zn^2+^), and other nutrients necessary for maintaining normal physiological functions of the human body.^23^, ^24^ It also contains a variety of antioxidant active ingredients, such as vitamin E, flavonoids, lipids, olefins, phenols, and aldehydes. Vitamin E is a well‐known antioxidant that breaks the lipid chain reaction.^22^ Flavonoids can also form a chelate with metal ions to inhibit the generation of free radicals and have the ability to scavenge free radicals and antioxidation.^25^ In a recent study, we tested the free radical scavenging activity and also proved that flavonoids possess obvious free radical scavenging activity.^26^ Moreover, we also confirmed that the intervention of *C. chinensis* can repair the reproductive system injury caused by acute Mn^2+^ impregnation in rats, reduce the MDA level of serum and testicular tissue, improve the SOD activity and T‐AOC level, and significantly improve the morphological structure of testicular tissues in rats with acute Mn^2+^ impregnation.^12^, ^27^ In the present study, we found that the activity of SOD, T‐AOC, and GSH‐Px was significantly increased in testis tissues after high dose of CcE treatment. These results indicate that CcE may inhibit the ROS level by increasing antioxidant enzyme activity, thereby inhibiting the mitochondria‐mediated cyt *c*/caspase‐3 signaling cascade apoptosis pathway. In summary, our study demonstrated that Mn^2+^‐induced testicular damage could be repaired by CcE, and the main factors responsible for the testicular protective effect were the antioxidant activity of CcE.

## CONFLICT OF INTEREST

The authors declare no potential conflict of interest.
